# Our Journey Through Advanced Therapies to Reduce Post-Infarct Scarring

**DOI:** 10.1007/s12015-021-10190-2

**Published:** 2021-05-22

**Authors:** Santiago Roura, Marta Monguió-Tortajada, Cristina Prat-Vidal, Carolina Gálvez-Montón, Antoni Bayes-Genis

**Affiliations:** 1 ICREC Research Program, Health Science Research Institute Germans Trias I Pujol, Can Ruti Campus, Badalona, Spain; 2 Heart Institute (iCor), Germans Trias I PujolUniversityHospital, Badalona, Spain; 3 CIBERCV, Instituto de Salud CarlosIII, Madrid, Spain; 4 Faculty of Medicine, University of Vic-Central University of Catalonia, Vic, Spain; 5 Institut D’Investigació Biomèdica de Bellvitge - IDIBELL, L’Hospitalet de Llobregat, Barcelona, Spain; 6 Cardiology Service, Germans Trias I Pujol University Hospital, Badalona, Spain; 7 Department of Medicine, Universitat Autonoma de Barcelona, Barcelona, Spain

“As you set out for Ithaka

hope your road is a long one,

full of adventure, full of discovery.”

Ithaka. C.P. Cavafy: Collected Poems 1975

We have read with interest the paper of Lin et al. in *Stem Cell Reviews and Reports* summarising the contributions of cardiac adipose tissue to treat myocardial infarction (MI) [1]. Meaningful advances in rapid reperfusion strategies and pharmacological management of patients with MI have reduced dramatically its mortality rates. Nevertheless, disease sequelae remain, including myocardial scarring ensuing heart failure. This has promoted a variety of innovative therapeutic endeavours to reduce infarct scars. A couple of decades ago, in the early days of the cardiac regeneration journey, cell-based therapy emerged as a promising option to regenerate post-infarcted myocardium through the implantation of pro-regenerative cells. In this context, we began to grow mesenchymal stromal cells (MSCs) in vitro, mostly from cardiac adipose tissue biopsies. These cells improved cardiac function, and reduced scar size in the MI murine model [2]. Moreover, by using peptide hydrogels, we were able to repopulate decellularised cardiac tissue (porcine myocardium or human pericardium) with MSCs to generate small (2 cm^2^) tissue engineering constructs or grafts with preserved macromechanical and micromechanical properties. The resulting three-dimensional (3-D) porous bioprostheses or bioimplants, which could also be engineered to include sophisticated electronic systems for online monitoring of myocardial tissue performance and repair, were valuable for limiting post-infarct sequelae in pigs [3–5].

In light of this promising experience, we then focused on manufacturing a clinical-size (12–16 cm^2^), engineered bioimplant by combining good manufacturing practice (GMP)-grade human Wharton's jelly-derived allogeneic MSCs and a decellularised human cadaveric pericardial matrix. Thus, over the past year, this novel advanced therapy medicinal product (ATMP), termed PeriCord, was completed and approved by the Spanish Agency of Medicines and Medical Devices for clinical use. Preliminary safety data from the first roll-in patient treated with PeriCord has already been reported in the context of the phase I PERISCOPE trial (the PERIcardial matrix with mesenchymal Stem Cells fOr the treatment of PatiEnts with infarcted myocardial tissue; EudraCT no. 2018-001964-49; clinicalTrials.gov identifier: NCT03798353), which is still enrolling patients [6].

Alternatively, we conceived and tested the Adipose Graft Transposition Procedure (AGTP), a surgical approach that smartly integrates cell therapy with cardiac tissue engineering. AGTP is based on the dissection of a vascularised flap of autologous pericardial adipose tissue to cover the scarred area, and limits myocardial remodelling by exerting beneficial effects on left ventricular function recovery. Interestingly, this strategy also avoids the use of non-autologous cells and ex vivo manipulation. A phase I clinical study (the AGTP-I trial, NCT01473433) has already proven its safety in humans [7], and a phase II-III multicentre clinical trial (the AGTP-II trial, NCT02798276) which is currently recruiting patients, will provide meaningful data on efficacy.

In present times, as Lin et al. convincingly point out, the expanded knowledge regarding the limited regeneration capacity of the adult human heart, and the short lifespan of delivered MSCs, have led the scientific community to reconsider the most suited strategies aimed at reducing myocardial injury. Thus, rather than focusing on the regeneration of an already carved scar and the differentiation capacities of MSCs, effort has shifted towards the modulation of the exacerbated inflammatory response that worsens cardiac tissue damage after MI, and using the immunoregulatory properties of the MSCs themselves and their secreted extracellular vesicles (EVs) to exploit this window of opportunity. In this context, we have recently provided new data on the design and features of 3-D biocompatible cell-free cardiac grafts for the local delivery of multifunctional cardiac adipose MSC-EVs, and their effects 6 days from implantation in pigs [8]. In specific, porcine MSC-EVs are powerful pro-angiogenic and immune modulatory agents, in terms of both proliferation and cytokine response abrogation (something not yet fully elucidated in swine), actively recruit pro-reparative allogeneic cells (thus they would be capable of promoting tissue repair by host or endogenous progenitor cells), and can be confined and locally administered, increasing the on-site EV amount. As a preliminary short-term functional description, implanted nanosized vesicles trigger neovascularisation and reduce the number of infiltrating macrophages and T cells. Indeed, we now expect to confirm whether this next generation of small-size decellularised pericardial matrix-based bioimplant comprising MSC-EVs attains long-term scar healing and cardiac function recovery, and can be clinically scalable and biomanufactured under GMP requirements to a novel allogeneic ATMP, as it has been achieved for PeriCord.

## Data Availability

Not applicable.
